# Validity and psychometric properties of the Severe Cognitive Rating Scale‐English version, among individuals with dementia

**DOI:** 10.1002/alz.087159

**Published:** 2025-01-09

**Authors:** Shreya Harikrishnan, Matan Soffer, Hiba Alhabbal, Amer M. Burhan, Sarah Colman, Li Chu, Simon Davies, Peter Derkach, Sarah Elmi, Philip Gerretsen, Ariel Graff‐Guerrero, Maria Hussain, Zahinoor Ismail, Donna Kim, Linda Krisman, Rola Moghabghab, Benoit H. Mulsant, Bruce G. Pollock, Vasavan Nair, Tarek K. Rajji, Soham Rej, Aviva Rostas, Lisa Van Bussel, Sanjeev Kumar

**Affiliations:** ^1^ University of North Carolina‐Chapel Hill, Chapel Hill, NC USA; ^2^ Centre for Addiction and Mental Health, Toronto, ON Canada; ^3^ Adult Neurodevelopment and Geriatric Psychiatry Division, CAMH, Toronto, ON Canada; ^4^ Institute of Medical Science, University of Toronto, Toronto, ON Canada; ^5^ Western University, London, ON Canada; ^6^ Ontario Shores Centre for Mental Health Sciences, Whitby, ON Canada; ^7^ University of Toronto, Toronto, ON Canada; ^8^ Ukrainian Canadian Care Centre, Toronto, ON Canada; ^9^ Department of Psychiatry, Temerty Faculty of Medicine, University of Toronto, Toronto, ON Canada; ^10^ Queen’s University, Kingston, ON Canada; ^11^ Hotchkiss Brain Institute, University of Calgary, Calgary, AB Canada; ^12^ McGill University, Montreal, QC Canada; ^13^ Toronto Dementia Research Alliance, University of Toronto, Toronto, ON Canada; ^14^ Temerty Faculty of Medicine, Division of Psychiatry, University of Toronto, Toronto, ON Canada; ^15^ Temerty Faculty of Medicine, University of Toronto, Toronto, ON Canada

## Abstract

**Background:**

Commonly used screening measures of cognitive function such as the Montreal Cognitive Assessment (MoCA) are not sensitive to assess cognitive function among individuals with severe cognitive impairment due to floor effect. The Severe Cognitive Impairment Rating Scale (SCIRS) was designed to assess cognitive function in those with severe cognitive impairment, however, psychometric properties of its English version have not been reported.

**Method:**

Using the existing data from StaN and tTED studies, floor and ceiling effects (percentage of minimal or maximal scores) of SCIRS and MoCA were examined, and the association between SCIRS and MoCA was evaluated.

**Result:**

Data from 141 participants (mean age = 78.7, 56% females) who completed either the SCIRS (n = 122) or MoCA (n = 80) were collected (n = 61 completed both). There was robust association between SCIRS and MoCA, supporting criterion validity of the SCIRS as a measure of cognitive function. SCIRS had a lower floor effect (13.1% minimal scores) as compared to the MoCA (27.5% minimal scores). Out of 22 participants with minimal scores on the MoCA, 16 participants completed the SCIRS with mean score of 9.8 (SD = 7.5).

**Conclusion:**

SCIRS appears to be a valid measure of cognitive function, showing better variance among individuals with severe cognitive impairment, as compared to MoCA.

